# Post-Hoc Analysis of Potential Correlates of Protection of a Recombinant SARS-CoV-2 Spike Protein Extracellular Domain Vaccine Formulated with Advax-CpG55.2-Adjuvant

**DOI:** 10.3390/ijms25179459

**Published:** 2024-08-30

**Authors:** Nikolai Petrovsky

**Affiliations:** 1 Vaxine Pty Ltd., Warradale, Adelaide 5046, Australia; nikolai.petrovsky@vaxine.net; 2 Australian Respiratory and Sleep Medicine Institute, Adelaide 5042, Australia

**Keywords:** COVID-19, SARS-CoV-2, vaccine, adjuvant, Advax, CpG, protection, correlate

## Abstract

SpikoGen^®^ vaccine is a subunit COVID-19 vaccine composed of an insect cell expressed recombinant spike protein extracellular domain formulated with Advax-CpG55.2™ adjuvant. A randomized double-blind, placebo-controlled Phase II clinical trial was conducted in 400 adult subjects who were randomized 3:1 to receive two intramuscular doses three weeks apart of either SpikoGen^®^ vaccine 25 μg or saline placebo, as previously reported. This study reports a post hoc analysis of the trial data to explore potential immune correlates of SpikoGen^®^ vaccine protection. A range of humoral markers collected pre- and post-vaccination, including spike- and RBD-binding IgG and IgA, surrogate (sVNT), and conventional (cVNT) virus neutralization tests were compared between participants who remained infection-free or got infected over three months of follow-up. From 2 weeks after the second vaccine dose, 21 participants were diagnosed with SARS-CoV-2 infection, 13 (4.2%) in the SpikoGen^®^ group and 8 (9%) in the placebo group. Those in the vaccinated group who experienced breakthrough infections had significantly lower sVNT titers (GMT 5.75 μg/mL, 95% CI; 3.72–8.91) two weeks after the second dose (day 35) than those who did not get infected (GMT 21.06 μg/mL, 95% CI; 16.57–26.76). Conversely, those who did not develop SARS-CoV-2 infection during follow-up had significantly higher baseline sVNT, cVNT, spike-binding IgG and IgA, and RBD-binding IgG, consistent with a past SARS-CoV-2 infection. SpikoGen^®^ further reduced the risk of re-infection (OR 0.29) in baseline seropositive (previously infected) as well as baseline seronegative participants. This indicates that while SpikoGen vaccine is protective in seronegative individuals, those with hybrid immunity have the most robust protection.

## 1. Introduction

Seroprevalence studies suggest more than 80% of the global population has already had a SARS-CoV-2 infection, indicating a high background level of natural immunity [1]. However, new immune-escape SARS-CoV-2 variants continue to circumvent both natural and vaccine-induced immunity, resulting in large waves of infection in many countries.

A variety of vaccines targeting the viral spike (S) protein are currently in use globally [2,3,4,5]. Our team designed a protein-based vaccine (SpikoGen^®^) built on experience developing vaccines against SARS [6,7] and MERS [8] coronaviruses. SpikoGen^®^ vaccine is based on the spike protein extracellular domain (ECD) sequence of the ancestral Wuhan-Hu-1 strain, modified by removing the furin cleavage site and expressed in insect cells using the baculovirus system [9].

Protein subunit vaccines generally have low immunogenicity. Hence, the SpikoGen^®^ vaccine is formulated with Advax-CpG adjuvant, a combination of delta inulin polysaccharide and the human toll-like receptor (TLR)-9 agonist, CpG55.2 (Vaxine, Australia). Advax-CpG adjuvant has previously been shown to enhance protective humoral and T cell responses in a variety of contexts [9,10,11]. For example, a SARS coronavirus vaccine based on the recombinant spike protein extracellular domain, formulated with Advax-CpG adjuvant, protected mice from a lethal dose of SARS virus and also prevented vaccine-enhanced disease caused by eosinophilic pneumonia [6,7]. A MERS vaccine based on a similar approach induced neutralizing antibodies and protected camels and alpacas against MERS coronavirus infection and shedding [8]. In mice, SpikoGen^®^ vaccine induced neutralizing antibodies and memory CD4+ and CD8+ T cells, and in immunized ferrets protected against lung infection [11]. In hamsters, SpikoGen^®^ vaccine prevented clinical disease and transmission to naïve co-housed sentinel animals [12,13]. Uncertainty remains regarding the mechanisms of COVID-19 vaccine protection. Serum and mucosal-neutralizing antibodies, memory B cells, and memory T cells have all been proposed as potential correlates of protection, with the paradox that spike antibody levels predict vaccine efficacy at a population level, but not at the individual level [14,15]. One challenge in establishing a potential correlation of vaccine protection is that the mechanism of protection may differ for different types of vaccine. Whereas one type of vaccine might protect through the induction of neutralizing antibodies, another vaccine may protect through the induction of anti-viral T cells. This emphasizes the importance of assessing potential correlates of protection for each individual vaccine, as they should not be assumed to always be the same. A Phase 2 trial of SpikoGen^®^ vaccine was undertaken in mid-2021 as previously reported [16]. Data on PCR-diagnosed COVID-19 infections reported by trial participants were collected during the trial and this plus antibody levels from stored sera was used to perform a post hoc analysis of potential correlates of SpikoGen^®^ protection.

## 2. Results

### 2.1. Evidence of Prior Infection in Trial Participants at Baseline

Just 11.5% of subjects were positive for N antibodies at baseline [16]. This may underestimate past SARS-CoV-2 infections as N antibody can become negative over time [17]. Instead, when positivity for any serum SARS-CoV-2 antibody, including binding IgG for N, S1, or RBD, binding IgA for S1 or RBD, or sVNT or pVNT, was used to identify participants with prior SARS-CoV-2 infection, 144/311 (46.3%) of the SpikoGen^®^ group was positive for at least one antibody and 37/89 (41.6%) of the placebo group. This suggests that just under half the study participants had already had a SARS-CoV-2 infection prior to their study entry in May 2021.

### 2.2. SARS-CoV-2 Infections in Trial Participants

**Figure 1:**
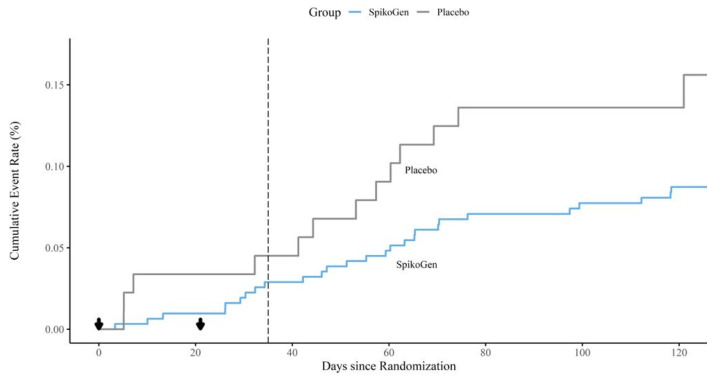
Kaplan Meir curve showing cumulative SARS-CoV-2 infections in SpikoGen^®^ and placebo groups. Black down arrows indicate the timing of the first and second vaccine or placebo dose. The dotted line represents two weeks post-second dose, the normal point from which infections counting towards COVID-19 vaccine efficacy are accrued.

Although SARS-CoV-2 infection was not a formal study endpoint, data on cases of PCR-confirmed SARS-CoV-2 infections was collected amongst study participants over three months of follow-up post-vaccination. The Kaplan Meir curve comparing infection rates in the SpikoGen^®^ and placebo groups over this period is shown in Figure 1. In the three weeks between the first and second dose, the SpikoGen^®^ vaccine group reported a 3.5-fold lower frequency of infection (3/311, 0.96%) than the placebo group (3/89, 3.37%), equating to an odds ratio of infection of 0.286 for the single-dose SpikoGen^®^ group. This was around May 2021, when alpha was still the dominant circulating variant. Delta replaced alpha as the dominant strain in June/July 2021, with a major wave of delta infections occurred just as most trial participants were receiving their scheduled second vaccine dose. From two weeks after the second vaccine dose, 21 participants were diagnosed with SARS-CpoV-2 infection, 13/311 (4.2%) in the SpikoGen^®^ group and 8/89 (9%) in the placebo group, equating to an odds ratio of infection in the SpikoGen^®^ group of 0.47.

### 2.3. Putative Immune Correlates of Protection

**Figure 2:**
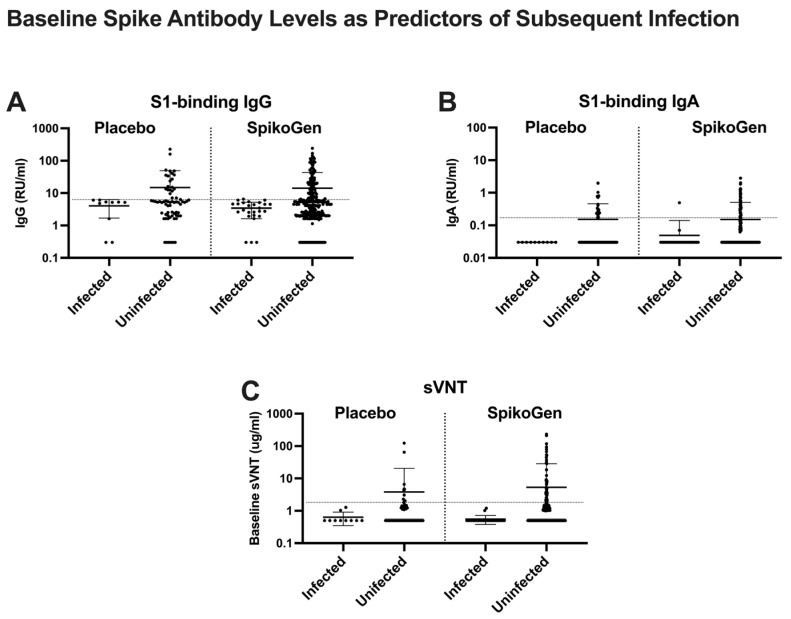
Baseline (Day 0) spike antibody levels (serum S1-binding IgG (**A**), S1-binding IgA (**B**), and surrogate virus neutralization test (sVNT) (**C**)) in participants who subsequently either did or did not have a documented SARS-CoV-2 infection during 3 months of follow-up. Mean plus standard deviation. The horizontal dotted line on each figure indicates the seropositive cutoff of each assay.

To explore whether baseline spike antibody levels at study entry (a marker of previous infection) influenced the risk of subsequent SARS-CoV-2 infection, we analyzed whether there were any differences in baseline antibody levels between those participants who reported PCR-confirmed infections during three months of study follow-up starting two weeks after the second dose (susceptible group) and those who did not report infections over this period (protected group). Irrespective of whether they were assigned to the placebo or active vaccine group, there was a clear inverse relationship between baseline spike antibody levels, whether measured by spike-binding IgG or IgA or sVNT and subsequent risk of SARS-CoV-2 infection (Figure 2).

Almost all SARS-CoV-2 infections occurred in those seronegative for spike antibody at baseline. This indicates that baseline spike seropositive participants (a marker of previous infection) even if in the placebo group, were protected against subsequent infection during the 3-month follow-up period. Notably, no trial participant with a baseline sVNT above the 2.5 μg/mL seropositive cutoff reported an infection during follow-up. Hence participants with high baseline spike antibody levels had likely had the most recent SARS-CoV-2 infections and thereby were protected by natural immunity even if randomized to the placebo group. The variant causing a recent infection would also be most likely to match the variants causing infections during the 3-month follow-up period, further explaining why these individuals were protected against infection.

**Figure 3:**
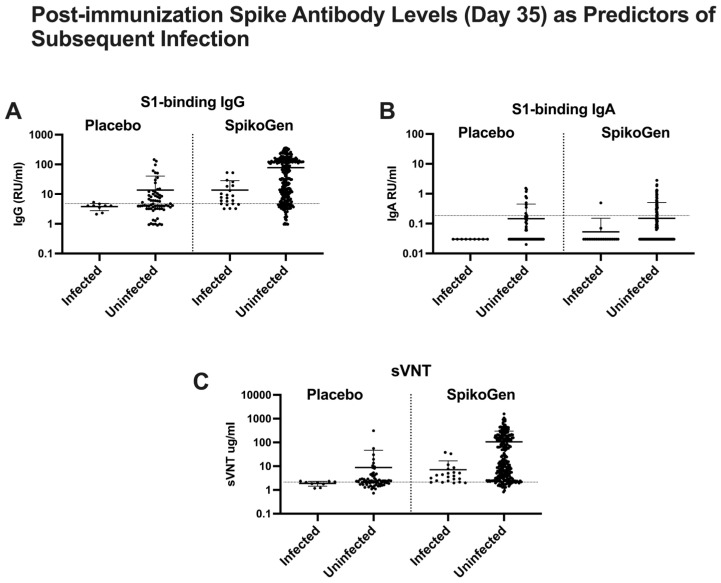
Spike antibody levels (serum S1-binding IgG (**A**), S1-binding IgA (**B**), and surrogate virus neutralization test (sVNT) (**C**)) measured two weeks after the 2nd SpikoGen^®^ or placebo dose (Day 35) in participants who subsequently either did or did not have a documented SARS-CoV-2 infection during 3 months of follow-up. Mean plus standard deviation. The horizontal dotted line on each figure indicates the seropositive cutoff of each assay.

We next looked at the relationship between antibody levels two weeks after the 2nd dose (day 35) and the risk of infection in the period starting two weeks after the 2nd dose and extending for three months post-vaccination. Susceptible individuals in the placebo group who subsequently became infected were all seronegative for all three types of serum spike antibodies at both baseline and day 35 (Figure 3). Most individuals in the SpikoGen^®^ vaccinated group had seroconverted to spike-binding IgG and sVNT two weeks after the second vaccine dose, although many remained seronegative for spike-binding IgA. Mean spike-binding IgG and sVNT levels two weeks after the 2nd dose were higher in the SpikoGen^®^ vaccinated group who remained uninfected versus those who became infected, but nevertheless most who became infected were in the group seropositive for spike-binding IgG and sVNT post-vaccination. This suggests that low seropositive spike-binding IgG in the range of 8–80 RU/mL or low sVNT in the range of 2.5–10.0 μg/mL post-vaccination did not predict protection against subsequent infection. Interestingly, seropositivity for spike-binding IgA was predictive of protection in immunized participants, with all those infected bar one being seronegative for IgA at Day 35, and all IgA seropositive participants bar one remaining uninfected during follow-up (Figure 3B).

The best day 35 predictor that separated those who subsequently became infected and those who did not was a sVNT > 2.5 μg/mL, a level above which no subjects in the SpikoGen^®^ or placebo groups became infected. Notably, 68.7% of subjects in the SpikoGen^®^ group had a sVNT > 2.5 μg/mL at day 35 versus just 30.7% in the placebo group [16].

### 2.4. Asymptomatic Infections in the Placebo Group between Day 0 and Day 35

To determine whether subjects in the placebo group might have had asymptomatic infections between day 0 and day 35, we looked for seroconversion in sVNT levels at day 35 in those in the placebo group who had not reported an infection between day 0 and 35. In the placebo group participants who did not report an infection between day 0 and 35, 10.1% were seropositive for sVNT (>2.5 μg/mL) at baseline, and this rose to 30.4% at day 21 and 33.3% at day 35, consistent with a high rate of undiagnosed infections in the placebo group during this short 5-week window up to day 35. In fact, the infection rate in the placebo group may have been even higher than this, as if sVNT seroconversion also included those who had a 4-fold increase in sVNT titer from baseline, then the sVNT seroconverted rate would have been 39.1% at day 21 and 69.6% at day 35. This suggested that many of the placebo group had undiagnosed SARS-CoV-2 infections over this 5-week period. These participants may have been undiagnosed because they were asymptomatic, were symptomatic and did not get tested, or were tested but delivered a false negative PCR result. Hence, while only 14.8% of the placebo group were diagnosed as infected during the follow-up period, the serological data suggests the true rate of infection in the placebo group may have been far higher than this. This is consistent with other studies suggesting at least 50% of SARS-CoV-2 infections are asymptomatic [18]. It was not possible to use spike antibody seroconversion in the vaccinated group to estimate the rate of asymptomatic infections in this group.

## 3. Discussion

Delta replaced alpha as the dominant variant at a time when many of the trial participants were scheduled to receive their 2nd SpikoGen^®^ dose. The cumulative infection curves of the vaccine and placebo groups temporarily came closer together around this time before separating again starting around two weeks after the 2nd dose (Figure 1). Hence, while a single SpikoGen^®^ dose was protective against the alpha variant, two doses were needed to protect against the delta variant, as also seen in animal studies. Back extrapolations based on serology predicted a SpikoGen^®^ efficacy after two doses of > 90% against the original ancestral SARS-CoV-2 strains [19]. Based on the high levels of sVNT seroconversion and seropositivity at day 35 in the placebo group, it seems a high proportion of placebo participants contracted delta infection over just this 5-week window. This suggests almost of the SpikoGen^®^ group would similarly have been exposed to delta infections during this same time, but with only 8.7% diagnosed with SARS-CoV-2 infection. This suggests that SpikoGen^®^ protection may have been even higher than calculated. Two weeks after the 2nd vaccine dose, 4.4% of the SpikoGen^®^ group and 10% of the placebo group were diagnosed with infection, equating to an odds ratio of symptomatic delta infection in the SpikoGen^®^ group of 0.46. This was similar to efficacy estimates in the SpikoGen^®^ Phase 3 combined per protocol plus nuclear antibody positive population, where infections were diagnosed in 0.56% of the SpikoGen^®^ group versus 1.46% of the placebo group from 14 days after the second dose, which translated to a vaccine efficacy of 64.36% (95% CI; 46.54 to 76.11) [19]. Notably, the Phase 3 trial also confirmed a highly significant SpikoGen^®^ vaccine efficacy of 77.5% in preventing severe COVID-19 disease.

An ongoing challenge is the identification of correlates of COVID-19 vaccine protection [20]. The World Health Organisation (WHO) and other regulatory bodies have been working to address standardization issues by making serological standards available [21,22]. However, the relationship between spike-binding antibodies post-vaccination and vaccine efficacy may only apply at the population level [14]. Even within a single vaccine trial, there is a high degree of overlap between antibody levels in vaccinated individuals who have breakthrough infections and those who do not [23]. This means that an individual’s spike antibody levels are a poor predictor of their personal risk of infection. Nevertheless, we were able to define cutoffs in spike antibody levels after vaccination, particularly spike-binding IgA, that predicted protection, with minimal infections occurring in participants who seroconverted to serum spike-binding IgA, post-immunization.

Baseline seropositive participants required just a single dose of SpikoGen^®^ to induce a robust rise in serum-spike antibodies. This is consistent with mRNA vaccine trials, where a single vaccine dose similarly induced a higher response in baseline seropositive participants [24], whereas most baseline seronegative participants only had a large increase in serum antibody levels after the 2nd dose. The strong amnestic B cell memory recall response after a single dose might thereby identify participants with prior SARS-CoV-2 infection (despite these participants being seronegative at baseline). Baseline seronegative participants after their 2nd SpikoGen^®^ dose achieved sVNT levels that, on average, were 3.3-fold higher than the mean level of baseline seropositive (convalescent) subjects. 

SARS-CoV-2 infects and transmits via the respiratory mucosa, making mucosal immunity of major importance alongside systemic immunity for protection. Normally, a parenterally administered vaccine would not be expected to induce mucosal immunity. However, SpikoGen^®^ vaccine induced a significant increase in serum spike binding IgA. Samples were not available to measure secretory IgA in this study, so it is unknown whether SpikoGen^®^ also increased secretory IgA. SpikoGen^®^-immunized ferrets had no recoverable virus in their nasal secretions three days after a SARS-CoV-2 challenge [11], raising the possibility that despite being administered parenterally, SpikoGen^®^ vaccine may still induce mucosal immunity which could help explain its beneficial effects in reducing the risk of transmission in animal studies.

While most focus has been on neutralizing antibodies, T cell immunity is also likely to be important for SARS-CoV-2 protection. T cell protection may also be more durable, with the SARS-CoV-2 antibody response relatively short-lived [25]. CD8 T cells play an important role in clearing virus-infected cells from the body [26] and synergize with cytotoxic antibodies, complement, and antibody-dependent cellular cytotoxicity to kill virus-infected cells. CoVac-1 is a T cell COVID-19 vaccine candidate composed of SARS-CoV-2 peptide epitopes combined with a Toll-like receptor two agonists emulsified in Montanide ISA5 that was shown to be safe and to induce T cell responses in a phase 1 clinical trial, although whether it will have protective efficacy is still unknown [27]. CpG55.2 as used as an adjuvant in SpikoGen^®^ is a potent TLR9 agonist that may assist in inducing CD8 T cell responses [28,29] and induced robust memory CD8 T cell responses in monkeys receiving a CMV vaccine [30]. Unfortnately, T cell responses are difficult to measure and compare between human studies given methodology differences and the general lack of T cell assay standardization [18,20].

Limitations of this study include the fact that the follow-up period was relatively short at three months, to allow unblinding so the placebo group could receive activeCOVID-19 vaccination. Furthermore, mucosal responses were unable to be studied. SpikoGen^®^ vaccine was based on Wuhan spike protein, with most study infections after the 2nd dose being caused by the heterologous delta variant so protect ion against the homologous Wuhan or alpha strains could not be directly assessed. Another study limitation was the lack of a serum sample taken at study completion, which would have been useful for assessing spike and N antibody seroconversion at the end of the 3-month follow-up period.

The COVID-19 pandemic emphasizes the importance of adjuvants such as Advax-CpG for achieving optimal responses to protein-based vaccines. While toll-like receptor (TLR)-9 was know to be the target of the CpG55.2 component of Advax-CpG only recently was it found that Advax particles bind and are internalized by dendritic cell-specific intercellular adhesion molecule-3-grabbing nonintegrin (DC-SIGN), a C-type lectin receptor expressed on dendritic cells [31]. While this is unlikely to be the full story behind the adjuvant action of Advax, it does help explain its anti-inflammatory properties [32]. For example, DC-SIGN has been shown to mediate the anti-inflammatory properties of IVIg [33], Salmonella Vi capsular polysaccharide [34], and even SARS-CoV-2 spike protein [35]. Furthermore, it may help explain the synergistic effects between Advax and CpG, as DC-SIGN mediates endosomal uptake of bacterial DNA by DCs, resulting in TLR9-dependent enhancement of B responses [36].

Notably, SpikoGen^®^ vaccine reduced SARS-CoV-2 infections in both baseline seropositive and seronegative participants. Protection in baseline seronegative participants correlated with the level of serum spike antibodies post-vaccination. While natural immunity due to prior infection provided robust protection against re-infection, nevertheless, SpikoGen^®^ further reduced the risk of re-infection by ~80% in seropositive participants. This shows that hybrid immunity from a combination of prior infection plus SpikoGen^®^ immunization provides extremely robust protection. Ironically, a vaccine that provides temporary sterilizing immunity that completely prevents infection, may not provide as good as long-term protection as a vaccine like SpikoGen^®^ that allows an early mild infection and thereby encourages the generation of robust hybrid immunity. The risk of COVID-19 infection has been reported to increase with the number of mRNA vaccine doses received [37,38], suggesting that mRNA vaccines may fail to induce or even act to prevent hybrid immunity. Those who have received multiple doses of mRNA vaccines exhibit a class switch to IgG4 antibodies that exhibit a reduced capacity to mediate antibody-dependent cellular phagocytosis, complement deposition, and other Fc-mediated effector functions critical for antiviral immunity, which may explain the increased infection risk once mRNA vaccine immunity wanes [39]. Notably, the issue of IgG4 after repeated boosters has only been reported with mRNA and, for example, was not seen with adenoviral boosters [37,38], and we have not seen it after protein-based boosters (manuscript in preparation). If so, a protein-based vaccine such as SpikoGen^®^ that still allows a single mild SARS-CoV-2 infections with no negative sequelae, thereby imparting hybrid immunity, may provide better long-term protection against SARS-CoV-2 morbidity and mortality [40]. 

Fortunately, the effect of COVID-19 vaccines on severe disease seems to be longer lasting than the effect on infection. It may not, therefore, be necessary for everyone to have multiple boosters once they have had a primary course although the optimal timing of COVID-19 boosters and who should receive them is still not clear. 

A key challenge for COVID-19 vaccines moving forward is the ongoing rapid evolution of the SARS-CoV-2 virus. This allows it to escape from pre-existing immunity and reinfect previously infected or vaccinated individuals. The high virus transmission rates observed in countries with high vaccine coverage, such as North America and Europe, suggest that the widely used mRNA vaccines may even select for vaccine-escape mutants [26]. A recent study showed that vaccine breakthrough infection with Omicron is common in the US, with the highest incidence among young adults, speculated to be due to age-related behavioral factors [27]. Recent new vaccine-escape variants include JN.1.7, KP.2, and KP.3 (together referred to as the FLiRT variants) [28]. This rapid evolution of SARS-CoV-2 indicates the need for ongoing research into more effective vaccines that can induce broadly cross-protective immunity against such new variants. This is something that adjuvanted protein-based vaccines may be better positioned to achieve than mRNA vaccines. Interestingly, a recent study suggested in those previously mRNA vaccinated that the best lung protection may be obtained using an intranasal protein-based booster [29]. We recently showed in a non-human primate study that two oral booster doses of SpikoGen^®^ vaccine after an initial 2-dose intramuscular course provides protection against a heterologous Omicron challenge [41]. This suggests the biggest future opportunities for protein-based COVID-19 vaccines such as SpikoGen^®^ may be as mucosal boosters.

Our study encountered no safety issues and found no evidence of any antibody-disease enhancement (ADE). ADE occurs when neutralizing antibodies, which protect against ADE, are lost first, leaving non-neutralizing binding antibodies that assist the virus to infect immune cells that express antibody-binding Fc receptors [30]. In dengue infections, Fc-mediated viral entry (FVE) enables the virus to enter and replicate in immune cells, resulting in profound immune disturbances and cytokine storms [33]. Viral entry into immune cells may also enable the virus to interfere with interferon production or antigen presentation [31]. ADE is most likely to occur when the vaccine antigen is a poor antigenic match to the key neutralizing epitopes on the infecting virus strain and sufficient time has passed for neutralizing antibody levels to have decayed, leaving only non-neutralizing binding antibodies still present. ADE has been a limiting factor in the development of other coronavirus vaccines, including those against feline coronavirus [32], the original SARS coronavirus [33] and the MERS coronavirus [34]. A recent study compared the effect of human SARS-CoV-2 convalescence on FVE into FcgRIIa-expressing HEK293 cells versus its virus-neutralizing activity [35]. It found a functional shift in convalescent plasma antibodies from combined neutralizing and FVE activity against the ancestral strain to just FVE activity and no neutralization activity against the Beta and Gamma variants. This would support the possibility that ADE could occur with SARS-CoV-2 in specific contexts where a non-neutralizing antibody with FVE activity is present, but a neutralizing antibody is absent. The possibility of ADE occurring in the setting of SARS-CoV-2 virus infection has been suggested by some in vitro and preclinical studies [30]. Although ADE has yet to be clearly confirmed to occur in the context of human SARS-CoV-2 infections or immunizations, a large epidemiological study identified double the risks of death, hospitalization, and other adverse sequelae in those experiencing a SARS-CoV-2 re-infection [31], which could thereby represent a potential signal of ADE. If ADE were to occur in relation to a COVID-19 vaccine, then this may manifest in a delayed rise in infections in the vaccinated arm as anti-spike antibody levels decayed, with this rise not being seen in the placebo arm. There was no evidence of this in the pattern of infections in our phase 2 trial; if anything, the frequency of these reduced over time in both groups and the infection curves continued to remain separate (Figure 1) even as spike antibody levels in the immunized group would have waned over time (even although these were not measured after day 35 in this study, some of the subjects later enrolled in a separate booster study about six months later and were found to have low or absent neutralizing antibody levels at that time [35]. This was also found to be the case in non-human primate studies where vaccine protection was maintained, and no evidence of ADE was observed in SARS-CoV-2 challenged SpikoGen^®^-immunized animals with no serum-neutralizing antibody but with residual non-neutralizing spike-binding antibody [36]. Notably, Advax-CpG adjuvant, as contained in the SpikoGen^®^ vaccine, was shown to prevent vaccine-enhanced disease when formulated with vaccine-enhanced disease-inducing SARS coronavirus vaccines [37].

In conclusion, this post hoc analysis of potential correlates of COVID-19 vaccine protection demonstrates the complexity of such analyses in a highly dynamic situation with new virus variants emerging and not all infections being symptomatic. The data showed that SpikoGen^®^ vaccine provided protection against the delta variant in both baseline seronegative and seropositive subjects. There is an ongoing need for more robust correlates of SARS-CoV-2 protection that predict protection at the individual level. While serum spike binding IgA levels post-vaccination appeared to be the best correlate of protection in our study, IgA rises post-vaccination may be a maker of a previous infection in otherwise seronegative individuals. T cell priming from the previous infection may be a key driver of protection in such individuals rather than their spike antibody levels. Teasing out such confounders is becoming increasingly difficult due to the fact that most people globally have now had one or more SARS-CoV-2 infections. Given this, confirmation of immune correlates of protection will need to rely on animal models where a lack of prior SARS-CoV-2 exposure can be assured.

## 4. Materials and Methods

### 4.1. Human Trial Samples

The Phase 2 trial involved 400 participants aged 18 years and older who were either healthy or had stable medical conditions. Participants were randomized 1:3 to receive saline placebo or SpikoGen^®^ vaccine as two intramuscular doses 21 days apart. The vaccine comprised 25 µg recombinant spike protein extracellular domain (ECD) together with Advax-CpG55.2 adjuvant (15 mg delta inulin + 0.15 mg CpG55.2). Sera were obtained at baseline (day 0) three weeks after the first dose (day 21) and two weeks after the second dose (day 35). The primary endpoints of the Phase 2 study were safety and immunogenicity, which have been previously reported [14]. Data on PCR confirmed SARS-CoV-2 infections was collected for three months following the second dose.

### 4.2. Antibody Measurements

Kits were used for assessing the levels of S1-binding (IgG ELISA-pishtazteb), S1-binding IgA (ELISA-Biosource) and surrogate virus neutralizing assay sVNT (ELISA-pishtazteb), as previously described [14].

### 4.3. Statistical Analysis

Continuous data were compared using *t*-test, and categorical data were assessed using Chi-square or Fisher exact test using GraphPad Prism version 9.4.0. The 95% CIs for GMC and GMFR were calculated based on the t-distribution of the log-transformed values and then back-transformed into the original scale at each time point for presentation. Hypothesis testing was two-sided, and *p* values of less than 0.05 were considered significant.

## Data Availability

Unidentified data is available upon request from the author.
